# Hidden Species Diversity was Explored in Two Genera of Catapyrenioid Lichens (Verrucariaceae, Ascomycota) from the Deserts of China

**DOI:** 10.3390/jof8070729

**Published:** 2022-07-13

**Authors:** Tingting Zhang, Xin Zhang, Qiuxia Yang, Xinli Wei

**Affiliations:** 1 State Key Laboratory of Mycology, Institute of Microbiology, Chinese Academy of Sciences, Beijing 100101, China; tingtingzhang813@163.com (T.Z.); monetax@163.com (X.Z.); yangqx@im.ac.cn (Q.Y.); 2 College of Life Sciences, University of Chinese Academy of Sciences, Beijing 100049, China; 3 College of Plant Sciences, Tibet Agricultural and Animal Husbandry University, Linzhi 860000, China

**Keywords:** catapyrenioid lichens, *Clavascidium*, new species, *Placidium*, taxonomy, Verrucariaceae

## Abstract

Verrucariaceae is the third-largest lichen family with high species diversity. However, this diversity has not been well-explored in China. We carried out a wide-scale field investigation in the arid and semi-arid regions of Northwest China from 2017 to 2021. A large number of lichen groups, especially those commonly distributed in deserts, were collected. Based on molecular phylogeny using ITS and nuLSU sequences by Bayesian and maximum likelihood analyses, combining morphological characters, seven taxa of catapyrenioid lichens in Verricariaceae were found in this study, including one genus (*Clavascidium*) and one species (*Clavascidium lacinulatum*) new to China; one genus (*Placidium*) new to the mainland of China; and four species (*Clavascidium sinense*, *Placidium* *nitidulum*, *Placidium* *nigrum*, and *Placidium* *varium*) new to science. It enriched our understanding of the high species diversity in Verrucariaceae and the lichen flora of Chinese arid and semi-arid deserts.

## 1. Introduction

The lichen family Verrucariaceae Eschw. is affiliated with Verrucariales, Eurotiomycetes, and Ascomycota, including 43 genera and 943 species [1]. Members of this family can colonize on various substrates, such as rock, soil, wood or bark, moss, and even other lichens [2]. Many species can tolerate harsh environments and participate in forming biological soil crusts (BSCs) in arid and semi-arid regions, such as catapyrenioid lichens [3].

The original catapyrenioid lichens are characterized by squamulose thalli, including *Catapyrenium* s.l. and *Endocarpon* Hedw. [4], between which *Catapyrenium* s.l. can be distinguished by simple ascospores and the absence of hymenial algae [5]. *Catapyrenium* s.l. generally referred to eight genera [6], i.e., *Anthracocarpon* Breuss, *Catapyrenium* Flot., *Clavascidium* Breuss, *Heteroplacidium* Breuss, *Involucropyrenium* Breuss, *Neocatapyrenium* H. Harada, *Placidium* A. Massal., and *Scleropyrenium* H. Harada; however, later research showed that the eight genera did not cluster into the same lineage [2] but scattered in two lineages and at least three groups, i.e., the *Endocarpon* group, *Placidium* group, and *Staurothele* group [2,4,7], among which only the *Placidium* group still represented the originally defined catapyrenioid lichens that have squamulose thalli, simple ascospores, and the absence of hymenial algae, including *Clavascidium*, *Heteroplacidium*, and *Placidium*, although sometimes, in *Heteroplacidium*, areolate to squamulose-areolate thallus also exist besides the smaller squamulose thallus [2]. *Placidium* refers to the members with squamulose thalli, usually well-developed medulla and a lower cortex, cylindrical or clavate asci, and laminal or marginal *Dermatocarpon*-type pycnidia [8]. Based on the thallus structure and asci type, *Clavascidium* and *Placidium* are delimited into different genera [9]. The characters such as medulla, asci shape, pycnidia position, and the presence or absence of rhizines are crucial taxonomic criteria, especially the medulla type, which is a significant character in the delimitation of the genera *Clavascidium*, *Placidium,* and *Heteroplacidium* [10]. The kind of medulla in Verrucariaceae is highly variable, which contains three types: prosoplectenchymatous, paraplectenchymatous, and mixed-type. The prosoplectenchymatous type is characterized by loosely interlaced hyphae with elongated cells [11], the paraplectenchymatous type is composed of tightly arranged and rounded cells, and the mixed-type medulla (“Mischtyp”) is composed of both rounded and elongated cells [12]. However, the variable medulla types generally occurred in *Clavascidium* and *Placidium*, not in *Heteroplacidium*, which only has the paraplectenchymatous medulla type.

In China, catapyrenioid lichens have not been well-studied; up to now, only two species of *Placidium* were reported, including *Placidium pilosellum* (Breuss) Breuss and *Placidium squamulosum* (Ach.) Breuss [13]. There is still a large space to explore the unknown species diversity in catapyrenioid lichens. Especially, those lichens are often the main components of biological soil crust in arid and semi-arid desert regions with important ecological functions, such as sand fixation by rhizines or lower surface and carbon fixations by photosynthesis [14,15]. Therefore, it has the important significance of exploring the catapyrenioid lichen species diversity for both taxonomy and ecology.

## 2. Materials and Methods

### 2.1. Taxon Sampling and Morphological Examination

**Figure 1:**
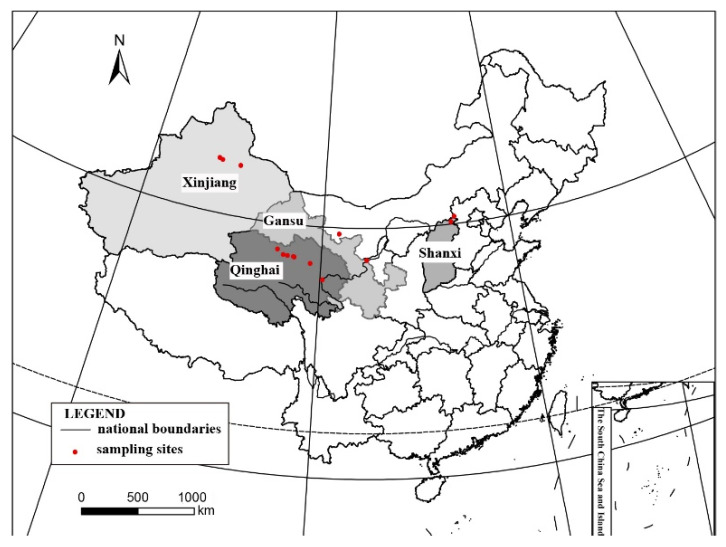
Collection sites. The detailed collection areas are marked in a solid red circle, and the corresponding four provinces involved are marked with varying degrees of a gray color.

About 3000 specimens were collected (Figure 1) from Northwest China in 2017–2021 and are preserved in the Lichen Section of Herbarium Mycologicum Academiae Sinicae, Beijing, China (HMAS-L), among which, 46 are *Clavascidium* and *Placidium* samples attracting our attention, because the two taxa have rarely been studied in China. The morphology and anatomy were examined using a MOTIC SMZ-168 stereomicroscope, a LEICA M125 dissecting microscope equipped with a Leica DFC450 camera, and a Zeiss Axio Imager A2-M2 equipped with Zeiss AxioCam MRC5 camera. The internal anatomy of the thallus was studied in sections of 10–15-µm thick cuts by a Leica CM1950 freezing microtome or by hand. Measurements were described as (a) b ± c (d), a = minimum value, b = mean value, c = standard deviation, and d = maximum value. Spot tests were performed using 10% KOH aqueous solution (K test) and Lugol’s iodine solution. Lichen secondary metabolites were examined using standardized thin-layer chromatography (TLC, solvent C) [16].

### 2.2. DNA Extraction, PCR, and Sequencing

The forty-six *Clavascidium* and *Placidium* specimens were extracted from DNA, followed by a modified CTAB method [17]. The PCR amplification of nrDNA ITS (internal transcribed spacer) was performed using the primers ITS4 and ITS5 [18] and nuLSU (large subunit) using the primers PRI1 and PRI2 [7]. Amplifications were performed in 25-µL volumes containing 12.5 µL 2 × Taq PCR MasterMix (Nanjing Vazyme Co., Ltd., Nanjing, China), 1 µL of each primer solution (10 µM), 9.5 µL ddH_2_O, and 1 µL dilutions (1:10) of genomic DNA. Amplifications were carried out in an ETC-811 plus thermal cycler (Beijing Eastwin Co., Ltd., Beijing, China), following conditions: an initial heating step for 5 min at 95 °C, followed by 35 cycles of 30 s at 94 °C, 30 s at 52 °C, and 1 min 30 s at 72 °C, with a final extension step of 10 min at 72 °C. The target PCR products were checked by electrophoresis on 1% agarose gels and then sequenced in SinoGenoMax Co., Ltd. (Beijing, China).

### 2.3. Sequence Alignment and Phylogenetic Analysis

A total of 160 DNA sequences including 61 new sequences (44 ITS, 17 nuLSU) were used in this study (Table S1) [2,4,7,19]. The new sequences generated for this study were deposited in GenBank. Twenty-nine accepted species previously sequenced, including 3 species of *Clavascidium*, accounting for 37.5% of the total 8 *Clavascidium* species, 19 species of *Placidium*, accounting for 57.6% of the total 33 *Placidium* species, and 7 species of *Heteroplacidium*, accounting for 58.3% the total 12 *Heteroplacidium* species, were included. *Placopyrenium* spp. were used as the outgroup. Raw sequences were assembled and edited with SeqMan [20] and then aligned using MAFFT v.7 [21]. The program Gblocks V0.19b [22,23] was used to remove ambiguously aligned sites. PAUP* v. 4.0 [24] was used for homogeneity testing (*p* > 0.05) before combining the two loci (ITS and nuLSU). All Maximum Likelihood and Bayesian analyses were performed using the GTR + I + G model selected by jModelTest 2 [25]. The randomized accelerated maximum likelihood (RAxML) analysis involving 1000 pseudoreplicates with RaxML v. 8.2.6 [26] was run on the Cipres Science Gateway (http://www.phylo.org, (accessed on 18 July 2011)). The Bayesian analysis performed using MrBayes v3.2.7 [27,28] with two parallel Markov chain Monte Carlo (MCMC), each using 5 million generations and sampling every 1000 steps, generating a 50% majority rule consensus tree after discarding the first 25% as the burn-in. TRACER v.1.7.2 [29] determined the burn-in value with effective sample sizes (ESS) higher than 200. All tree files were visualized with FigTree v.1.4.3 (http://tree.bio.ed.ac.uk/software/figtree, (accessed on 28 August 2014)). The clades with bootstrap (BP) values above 75 or posterior probability (PP) values above 0.95 were considered highly supported.

## 3. Results

### 3.1. Phylogenetic Analysis

**Figure 2:**
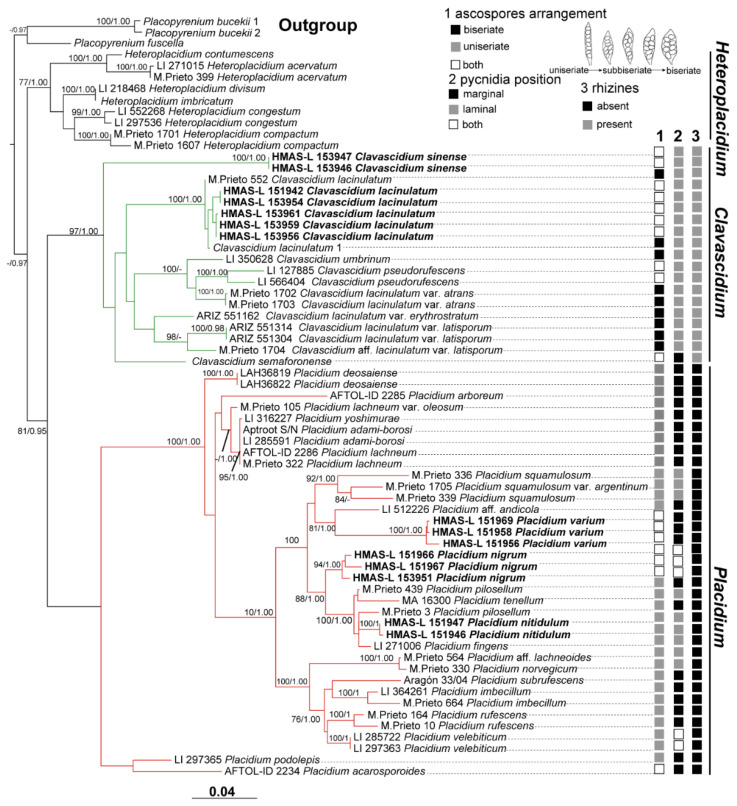
The RAxML tree is based on the concatenated ITS + nuLSU data set representing both ML and BI trees. The number in each node represents bootstrap support (BS) and posterior probability (PP) values. BS values ≥ 75 and PP values ≥ 0.95 were plotted on the branches. The taxa in bold indicate that these sequences were newly generated for this study. Green and red clades show *Clavascidium* and *Placidium*, respectively. The character status of 1 ascospores arrangement, 2 pycnidia position, and 3 rhizines are listed at the right of the tree, corresponding to each sample of *Clavascidium* and *Placidium*. Scale in 0.04 substitution per site.

A total of 61 sequences newly obtained in this study were used in the single-locus phylogenetic tree construction; among which, 30 sequences of the ITS and nuLSU generated from 15 specimens representing 5 species (7 *Clavascidium* specimens and 8 *Placidium* specimens) were combined for constructing the concatenated tree Figure 2. The concatenated matrix included 1204 variable positions (448 ITS and 756 nuLSU) after excluding ambiguous regions. The aligned matrix contained 1204 nucleotide position characteristics for the complete data set of 67 members. The concatenated BI phylogenetic tree and two single-gene-locus RAxML trees including 44 and 16 samples are shown in Figures S1–S3. *Placidium*, *Clavascidium*, and *Heteroplacidium* all formed monophyletic branches. Both the ML and BI phylogenetic trees produced similar topologies. There are four new species: *Clavascidium sinense* sp. nov., *Placidium nitidulum* sp. nov., *Placidium nigrum* sp. nov., and *Placidium varium* sp. nov. are well-supported.

### 3.2. Taxonomy

A key to the species of Clavascidium and Placidium is listed in Table 1.

***Clavascidium*Breuss**, Annln naturh. Mus. Wien, Ser. B, Bot. Zool. 98 (Suppl.): 41, 1996

The genus *Clavascidium* is characterized by squamulose thallus, the presence of rhizines, and clavate to (sub) cylindrical asci containing biseriate ascospores. Gueidan [2,9] introduced *Clavascidium* as a sister genus of *Placidium* based on morphological characters and a phylogenetic analysis, between which *Clavascidium* has clavate asci with biseriate ascospores, whereas *Placidium* has cylindrical asci with uniseriate ascospores. However, uniseriate or both biseriate and uniseriate ascospores were also reported [30].

**Figure 3:**
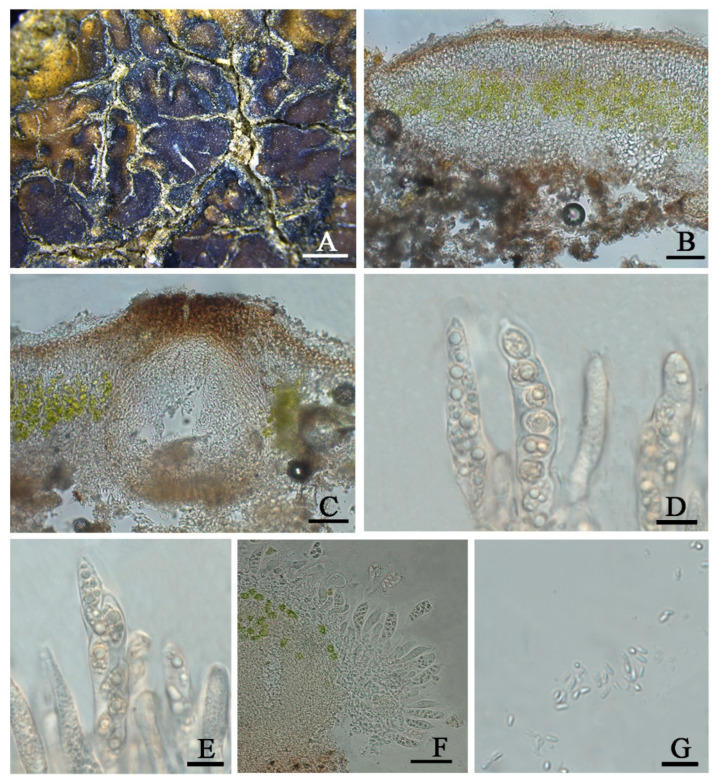
The thallus habit and the anatomic structure of *Clavascidium lacinulatum* (HMAS-L 153962). (**A**). Squamulose thallus. (**B**). Transversal section of thallus. (**C**). Immersed perithecium. (**D**,**E**). Uniseriate ascospores in cylindrical asci. (**F**). Biseriate ascospores in clavate asci. (**G**). Conidia. Bars: (**A**) =2 mm, (**B**,**C**) =50 µm, (**D**,**E**) =10 µm, (**F**) =50 µm, and (**G**) =10 µm.

***Clavascidium lacinulatum*** (Ach.) M. Prieto, in Prieto et al., Am. J. Bot. 99 (1): 28, 2012 (Figure 3)

≡ *Endocarpon hepaticum* var. *Lacinulatum* Ach Lich. univ.: 299, 1810.

**Description**: Thallus squamulose, terricolous, 300 µm thick; lobes 1–4 mm wide, roundish to deeply lobed, contiguous, rarely overlapping; upper surface dark brown, dull; lower surface pale ± rhizines; epinecral layer transparent, up to 15 µm thick; upper cortex paraplectenchymous, 50–70 µm thick, cells 4 × 10 µm in diam.; uppmost layer brown, 25–30 µm thick; photobiont layer 50–100 µm thick, algal cells 6–10 µm in diam; medullar tissue paraplectenchymous; lower cortex not delimited from the medulla, 40 µm thick, paraplectenchymous, composed of irregularly arranged roundish cells up to 7.5 µm in diam. Rhizohyphae hyaline, 4–6 µm wide. Pycnidia laminal, *Dermatocarpon*–type, immersed, subglobose, light brown, conidia oblong–ellipsoid to bacilliform, 1–1.3 × 3–3.7 µm in size.

Perithecia immersed, broadly pyriform (150 × 180 µm) to subglobose (up to 230 µm) wide; perithecia wall bright, 30–35 µm thick; hymenium bright, 35–45 µm thick; involucrellum brown, 42–44 µm thick; pyriphyses 35–40 × 2.5–3 μm; asci cylindrical to clavate, 14–18 × 40–52 µm, ascospores 8 per ascus, uniseriate to biseriate, ellipsoid to fusiform to ovoid, 5–7 × 10–12 µm.

**Chemistry**: All the spot tests were negative, and no substances were detected by TLC.

Habitat and distribution: These species grow on the surface of sandy soil in the semi-arid and arid region of Northwest China, located in the open areas with sun exposure. The surrounding environment is characterized by interlace of meadows, fixed undulating sand dunes under shrubs, and the Gobi Desert, with the elevations greatly varying from 923 to 3175 m. It distributes worldwide [30] and is new to China.

Specimens examined: CHINA. SHANXI: Datong City, Yanggao County. 40.98° N 113.88° E, 1264 m alt., on the sand, 14 April 2021, X. Qian & T.T. Zhang 20210273 (HMAS–L 153955), 20210274 (HMAS–L 153956), 20210283 (HMAS–L 153957); 40°25′51″N 113°44′19″E, 923 m alt., on the sand, 15 April 2021, X. Qian & T.T. Zhang 20210299 (HMAS–L 153962), 20210300 (HMAS–L 153958), 20210302 (HMAS–L 153959), 20210306 (HMAS–L 153960), 20210307 (HMAS–L 153961). GANSU: Baiyin City, Jingtai County. 37°19′41″ N 104°35′37″ E, 1524 m alt., on the sand, 21 October 2020, X. Qian et al. 20201529 (HMAS–L 153781). QINGHAI: Haixi Mongolian and Tibetan Autonomous Prefecture, Delhi City. 37°23′02″ N 97°08′44″ E, 3041 m alt., on the sand, 24 April 2021, X.L. Wei & T.T. Zhang 20210436 (HMAS–L 151942); 37°24′29″ N 96°30′43″ E, 3175 m alt., on the sand, 24 April 2021, X.L. Wei & T.T. Zhang 20210499 (HMAS–L 153954); Hainan Tibetan Autonomous Prefecture, Guinan County. 35°40′54″ N 100°15′08″ E, 1606 m alt., on the sand, 16 October 2020, X. Qian et al. QX20200026 (HMAS–L 153953).

**Notes**: This species is widespread in the desert regions of China as a crust (Figure 1).Our specimens are well-clustered with *Cl. lacinulatum* in the phylogeny [4] (Figure 2 and Figures S1–S3) and, also, with a high consistency of the phenotype described by Nash et al. [30]. Although this species is variable in the external morphology of thallus and squamules, several important taxonomic characters such as laminal pycnidia, oblong-ellipsoidal to subcylindrical conidia, and rhizines are constantly present. Breuss described uniseriate ascospores in this species [31]; however, we found this character is variable (Figure 3).

**Figure 4:**
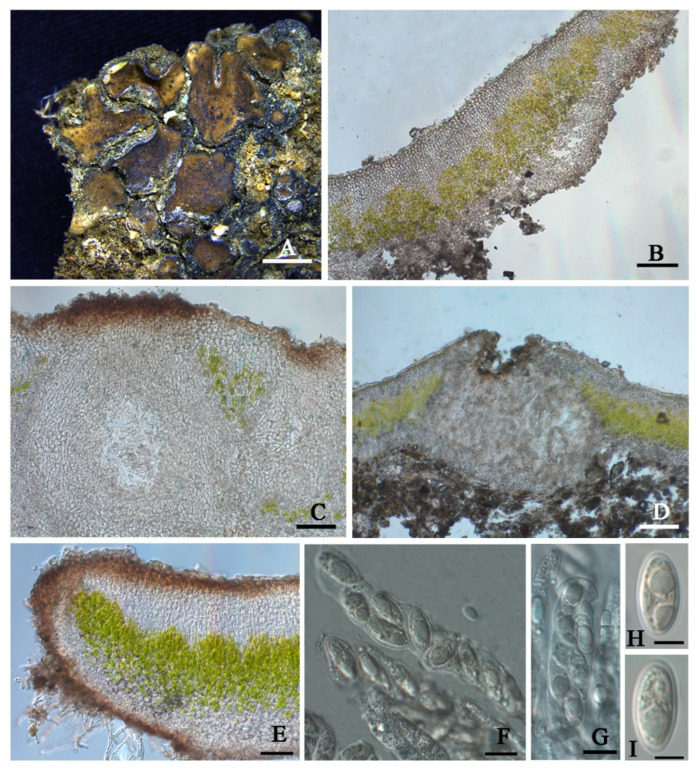
The thallus habit and the anatomic structure of *Clavascidium sinense* sp. nov (holotype HMAS-L 153946). (**A**). Squamulose thallus and immersed apothecia with the appearance of black spots. (**B**). Transversal section of thallus. (**C**). Perithecium. (**D**). Pycnidium. (**E**). Thallus section. (**F**). Uniseriate ascospores in cylindrical asci. (**G**). Biseriate ascospores in clavate ascus. (**H**,**I**). Ascospores. Bars: (**A**) =100 µm, (**B**) =100 µm, (**C**) =50 µm, (**D**) =2 mm, (**E**) =50 µm, (**F**,**G**) =10 µm, and (**H**,**I**) =5 µm.

***Clavascidium* *sinense*** T.T. Zhang & X.L. Wei, **sp. nov.** (Figure 4)

Fungal Names No.: FN571022

**Etymology:** The epithet ‘*sinense*’ refers to the Chinese distribution of this species.

**Typus:** CHINA. SHANXI: Datong City, Yanggao County. 40°25′28″ N 113°46′07″ E, 1177 m alt., on soil, 14 April 2021, X. Qian & T.T. Zhang 20210245 (holotype, HMAS–L 153946, ITS ON712842, nuLSU ON712829).

**Diagnosis:** It is characterized by the co-existence of both uniseriate and biseriate ascospores in asci, laminal pycnidia, and mixed-type medulla.

**Description:** Thallus squamulose, terricolous; lobes 1.5–4 mm wide, (194) 255 ± 32 (314) µm thick, deeply lobed, contiguous, rarely overlapping, full appressed to the substrate or with raised margins free from the substrate; upper surface medium to dark brown, dull; lower surface pale ± rhizines; epinecral layer often absent, if present up to 10 µm thick; upper cortex paraplectenchymatous, (57) 81 ± 19 (109) µm thick, cells (7.8) 9.9 ± 1.7 (12.6) µm in diam., uppmost layer bright brown, 15–25 µm thick; algal layer (70) 107 ± 24 (157) µm thick, composed of rounded cells (7.4) 9.5 ± 1.6 (12) µm in diam., globose to sub-globose; medulla mixed type, 60–100 µm thick; lower cortex not clearly delimited from the medulla, transparent, outer layer of lower cortex bright brown, (37) 51 ± 8.5 (59) µm thick, composed of rounded cells (7.2) 8.2 ± 0.9 (10.2) µm diam. Rhizohyphae 4–5 µm thick, colorless. Pycnidia laminal, *Dermatocarpon*-type, subglobose, light brown, conidia oblong-ellipsoid to bacilliform, (3.0) 3.4 ± 0.29 (3.7) × (1.26) 1.4 ± 0.18 (1.75) µm.

Perithecia laminal, immersed, subglobular, up to 400 µm in diam.; perithecia wall 40–55 µm thick; hymenium pale brown, 65–100 µm thick; involucrellum brown, 38–50 µm thick; pyriphyses 30–40 µm long; asci (sub)cylindrical to clavate, 64–84 × 12–15 µm, 8 spored, ascospores uniseriate to biseriate, hyaline, narrow ellipsoid, (10.2) 13 ± 1.2 (13.9) × (4.8) 6.3 ± 0.7 (7.3) µm.

**Chemistry:** All the spot tests were negative, and no substances were detected by TLC.

**Habitat and distribution:** On the surface of a broken great wall built by clay in the semi-arid region of Northwest China. It has been known only in China up to now.

Additional specimens examined: CHINA. SHANXI: Datong City, Yanggao County. 40°25′28″N 113°46′07″E, 1177 m alt., on soil, 14 April 2021, X. Qian & T.T. Zhang 20210246 (HMAS-L 153947).

**Notes:** Biseriate and uniseriate ascospores in asci are generally diagnostic characters of *Clavascidium* and *Placidium*, respectively [2,9]. However, uniseriate or both biseriate and uniseriate ascospores in asci were also found in *Clavascidium* [30], including *Cl. sinense*, indicating ascospores arrangement is a continuously changing character between these two closely related genera in phylogeny. The phylogenetic analysis clearly supported *Cl. sinense* obviously separated from other *Clavascidium* species with both types of ascospores arrangements, such as *Cl. lacinulatum*, *Cl. pseudorufescens* (Breuss) M. Prieto, and *Cl. semaforonense* (Breuss) M. Prieto. However, *Cl. sinense* has mixed-type medulla, distinct from *Cl. lacinulatum* with paraplectenchymous medulla and *Cl. pseudorufescens* with filamentous-hyphae medulla [30]. *Cl. sinense* has laminal pycnidia, but *Cl. semaforoense* has marginal pycnidia, which is an exception within the genus *Clavascidium* [8]. The phylogenetic trees (Figure 2 and Figures S1–S3) provided further support, indicating *Cl. semaforoense* formed a separate clade, far from all the other species of *Clavascidium*. However, the new species *Cl. sinense* situated in the outermost clade, and *Cl. semaforoense* situated in the secondary outermost, indicating that laminal or marginal pycnidia is not an absolutely unchanging character within this genus but can be used in delimitation species. There are three *Clavascidium* species absence of DNA sequences: *Clavascidium antillarum* (Breuss) Breuss, *Clavascidium imitans* (Breuss) M. Prieto, and *Clavascidium krylovianum* (Tomin) M. Prieto. However, *Cl. sinense* has distinct traits compared with these three species, for example, *Cl. antillarum* is characterized by dark brown to black lower surface and rhizines [5], while *Cl. sinense* is characterized by a pale lower surface and rhizines. *Cl. imitans* and *Cl. krylovianum* can be distinguished by the morphology of medulla; the former medullary hyphae divide into many short and swollen cells, but the latter medullary hyphae are filamentous [5]; in comparison, *Cl. sinense* has a mixed-type medulla.

***Placidium*** A. Massal. Symmict. Lich.: 75, 1855

The genus *Placidium* has squamulose thallus, laminal or marginal *Dermatocarpon*-type pycnidia, cylindrical asci, uniseriate ascospores, and rhizohyphae [8]. Two *Placidium* species have been reported from Taiwan by Dr. Aptroot [13]. This genus is firstly reported from Mainland China in this study, and we found some additional morphological characters in *Placidium* such as a glossy upper surface and aggregated pycnidia, which can be used to distinguish some species.

**Figure 5:**
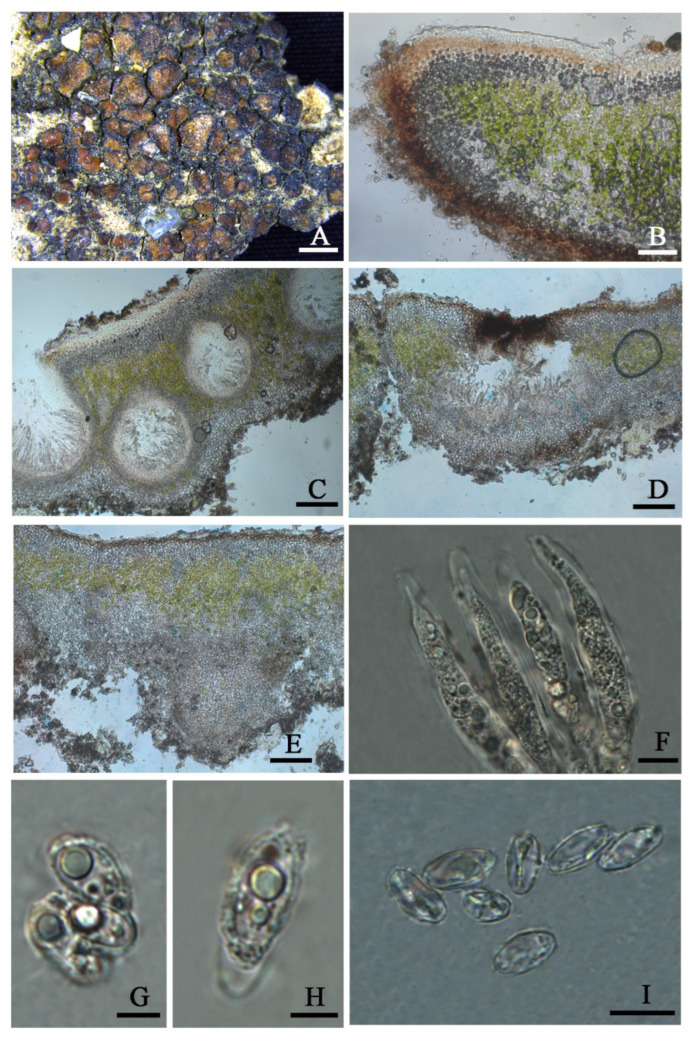
The thallus habit and the anatomic structure of *Placidium nitidulum* sp. nov (holotype HMAS-L 151947). (**A**). Squamulose thallus. (**B**). Transversal section of thallus. (**C**). Immersed perithecia. (**D**). Aggregated pyrithecia. (**E**). Pycnidium. (**F**). Cylindrical asci with uniseriate ascospores. (**G–I**). Ascospores. Bars: (**A**) =2 mm, (**B**) =50 µm, (**C–E**) =100 µm, (**F**) =10 µm, (**G**,**H**) =5 µm, and (**I**) =10 µm.

***Placidium nitidulum*** T.T. Zhang & X.L. Wei **sp. nov.** (Figure 5)

Fungal Names No.: FN571023

**Etymology:** The epithet ‘*nitidulum*’ refers to the glossy upper surface of the thallus in this species.

**Typus:** CHINA. QINGHAI: Haixi Mongolian and Tibetan Autonomous Prefecture, Wulan County. 36°57′31″ N 98°54′03″ E, 3314 m alt., on the sand, 25 April 2021, X.L. Wei & T.T. Zhang 20210560 (holotype, HMAS–L 151947, ITS ON712844, nuLSU ON712835).

**Diagnosis**: It is characterized by a glossy upper surface, tiny lobes, and a thick algal layer.

**Description:** Thallus squamulose; terricolous; lobes roundish, tiny, 0.7–2 mm wide, (356) 442 ± 55 (523) µm thick; contiguous to densely aggregated, tightly adnate to the substrate; upper surface medium brown, glossy, brown rimmed; lower surface pale to pale yellow; epinecral layer transparent, (33.4) 35.4 ± 1.6 (37.5) µm thick; upper cortex (41) 58 ± 15 (78) µm thick, paraplectenchymatous, cells 8–10 µm in diam.; uppermost layer pale yellow to light brown, 33–36 µm thick; algal layer (104) 159 ± 34 (195) µm thick, algal cells globular, (7.3) 7.8 ± 0.5 (8.7) µm in diam. dispersed over the whole medulla; medulla zone not clear; lower cortex not delimited from the medulla, (42) 63 ± 16 (88) µm thick, composed of densely aggregated globular cells, cells (8.4) 10.6 ± 1.5 (12.6) µm in diam. Rhizohyphae (4.0) 5.2 ± 0.7 (5.9) µm thick, colorless. Pycnidia laminal, immersed, *Dermatocarpon*-type, immature, subglobular, 250 µm in dim., conidia not seen.

Perithecia laminal, immersed, occasionally aggregated, narrowly pyriform, up to 300 µm wide; perithecia wall grey, 28–42 µm thick; hymenium pale yellow, 45–75 µm thick; involucrellum brown or absence, 68–90 µm thick; paraphyses branched, 35–55 × 1.5–2.5 µm; asci cylindrical, 45–65 × 5–8 µm, 8 spored, uniseriate, ascospores hyaline, narrow ellipsoid, (8.4) 8.7 ± 1.5 (11.5) × (5.1) 6.1 ± 0.5 (7.0) µm; paraphyses well-developed.

**Chemistry:** All the spot tests were negative, and no substances were detected by TLC.

**Ecology and distribution:** On the surface of sandy soil in high altitude areas of the Qinghai–Tibet plateau. It has been known only in China up to now.

Additional specimens examined: CHINA. QINGHAI: Haixi Mongolian and Tibetan Autonomous Prefecture, Delhi City. 37°23′02″ N 97°08′44″ E, 3041 m alt., on the sand, 24 April 2021, X.L. Wei & T.T. Zhang 20210421 (HMAS-L 151962); Wulan County. 36°57′31″ N 98°54′03″ E, 3314 m alt., on the sand, 25 April 2021, X.L. Wei & T.T. Zhang 20210552 (HMAS–L 151946).

**Notes:** This new species can be easily recognized by its glossy appearance of the upper surface and tiny lobes, which is very distinctive and different from all the other known *Placidium* species. The medulla zone is obscure due to being fully covered by the thick algal layer, so the lower cortex is difficult to delimit from the medulla, similar to *Placidium tenellum* (Breuss) Breuss in this character. However, the upper surface of *Pl. tenellum* is matt, the algal layer is thinner ((40) 93 ± 24 (155) µm), and the pycnidia is much broader (up to 500 µm). The distribution is more frequent in coastal areas [8]. Based on the phylogenetic trees (Figure 2 and Figures S1–S3), the new species is close to *Placidium fingens* (Breuss) Breuss, *Pl. pilosellum*, and *Pl. tenellum*, among which the first two are more intimate than the last one to the new species in phylogeny; however, the lobe widths of *Pl. fingens* and *Pl. pilosellum* (up to 6 mm) are nearly three times the new species (0.7–2 mm). *Pl. nitidulum* is closer to *Pl. fingens*, both of which have laminal pycnidia, while the species *Pl. pilosellum* within this subclade with a little far distance has marginal pycnidia. *Pl. nitidulum* has more slender asci (45–65 × 5–8 µm) than the species *Pl. pilosellum* (70–90 × 10–15 µm). Additionally, *Pl. nitidulum* has squamules with a smooth margin, but *Pl. pilosellum* has squamules with hairy margins [8].

**Figure 6:**
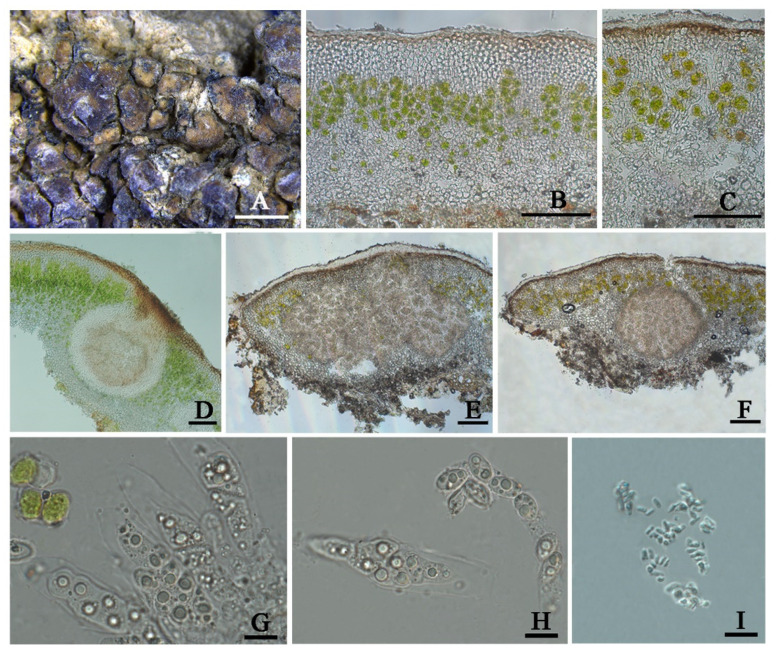
The thallus habit and the anatomic structure of *Placidium nigrum* sp. nov (HMAS-L 153950). (**A**). Squamulose thallus. (**B**,**C**). Thallus section. (**D**). Immersed perithecium. (**E**,**F**). *Dermatocarpon*-type pycnidia. (**G**,**H**). Asci with ascospores. (**I**). Conidia. Bars: (**A**) =2 mm, (**B**,**C**) =100 µm, (**D**) =200 µm, (**E**,**F**) =100 µm, and (**G**–**I**) =10 µm.

***Placidium******nigrum*** T.T. Zhang & X.L. Wei, **sp. nov.** (Figure 6)

**Fungal Names No.:** FN571024

**Etymology:** The epithet ‘*nigrum*’ refers to the surrounding black area due to the aggregation of abundant pycnidia of this species.

**Typus:** CHINA. QINGHAI: Haixi Mongolian and Tibetan Autonomous Prefecture, Dachaidan Region. 37°52′11″ N 95°15′54″ E, 3515 m alt., on the sand, 25 April 2021, X.L. Wei & T.T. Zhang 20210516 (holotype, HMAS–L 151944, ITS ON712851).

**Diagnosis**: It is characterized by both laminal and marginal pycnidia, with abundant aggregation forming into the surrounding black area.

**Description:** Thallus squamulose, terricolous; lobes 1–3 mm wide, (211) 336 ± 81 (462) µm thick, tumid roundish to lobate, scattered or contiguous to densely aggregated, tightly adnate to the substrate or with slightly raised margins; upper surface pale to medium brown, dull, pruinose; epinecral layer transparent, (12) 20.4 ± 7.2 (33) µm thick; upper cortex paraplectenchymatous, (31) 52 ± 11 (70) µm thick, cells (8.5) 10.5 ± 1.9 (13.8) µm in diam., uppermost layer brown, 8–12 µm thick; algal layer (37) 67 ± 14 (85) µm thick, composed of spherical cells, cells (7.4) 13.3 ± 2.9 (17.6) µm in diam., globose to subglobose, in clusters of 1–3 cells; medulla mixed type, (37) 69 ± 17 (102) µm thick; lower cortex delimited from the medulla, hyaline, (32) 44 ± 9.8 (73) µm thick, composed of spherical cells, cells (8.1) 9.8 ± 1.4 (12.4) µm in diam. Rhizohyphae (3.3) 4.4 ± 0.77 (5.7) µm thick, colorless. Pycnidia laminal and marginal, abundant, aggregated into all-round black area, immersed, *Dermatocarpon*-type, subglobular to irregular, up to (230) 274 ± 33 (317) µm wide; conidia bacilliform, (2.7) 3 ± 0.39 (3.5) × (0.96) 1.3 ± 0.29 (1.89) µm.

Perithecia laminal, immersed, subglobular, 320 × 350 µm; perithecia wall bright, 28–50 µm thick; hymenium 70–85 µm thick; involucrellum brown, 43–55 µm thick; periphyses 40–55 µm long; asci cylindrical to clavate, 45–65 × 9–18 µm, 8 spored, uniseriate to (sub)biseriate, ascospores hyaline, ellipsoid, (8.4) 9.9 ± 1 (11.5) × (4.0) 5.7 ± 0.9 (8.0).

**Chemistry:** All the spot tests were negative, and no substances were detected by TLC.

**Habitat and distribution:** On the surface of sandy soil in the semi-arid and arid region of Northwest China and the Qinghai–Tibet plateau. The distribution range is relatively wide, from low altitude to high altitude (438–3515 m alt.). The environment in which they grow is also more varied, including naturally formed bush bottoms and artificial vegetation fix sand forests (i.e., *Ammopiptanthus mongolicus* (Maxim. ex Kom.) Cheng f. and *Populus alba* var. *pyramidalis* Bge.), and the Gobi Desert, common in dry and open habitats. It has been known only in China up to now.

Additional specimens examined: CHINA. QINGHAI: Haixi Mongolian and Tibetan Autonomous Prefecture, Dachaidan Region. 37°52′11″ N 95°15′54″ E, 3515 m alt., on sand, 25 April 2021, X.L. Wei & T.T. Zhang 20210535 (HMAS–L 153951), 20210522 (HMAS–L 151945), 20210523 (HMAS–L 151965), 20210524 (HMAS–L 151966), 20210534 (HMAS–L 153950); Delhi City. 37°23′02″ N 97°08′44″ E, 3041 m alt., on the sand, 24 April 2021, X.L. Wei & T.T. Zhang 20210461 (HMAS–L 151963); Hainan Tibetan Autonomous Prefecture, Guinan County. 35°40′54″ N 100°15′08″ E, 1606 m alt., on the sand, 16 October 2020, X. Qian et al. 20201384 (HMAS–L 153732). GANSU: Baiyin City, Jingtai County. 37°25′06″ N 104°34′56″ E, 1591 m alt., on the sand, 21 October 2020, X. Qian et al. 20201486 (HMAS–L 153786). XINJIANG: Changji Hui Autonomous Prefecture, Fukang County. 44°22′59″ N 87°52′34″ E, 438 m alt., on the sand, 10 May 2021, X. Qian & T.T. Zhang 20210666 (HMAS–L 151952); Qitai County. 44°13′32″ N 90°02′32″ E, 723 m alt., on the sand, 10 May 2021, X. Qian & T.T. Zhang 20210746 (HMAS–L 151941); Wujiaqu City. 44°29′54″ N 87°28′21″ E, 408 m alt., on the sand, 9 May 2021, X. Qian & T.T. Zhang 20210606 (HMAS–L 151967). INNER MONGOLIA: Alxa Right Banner, 39°28′24″ N 101°04′03″ E, 1564 m alt., on sand, 22 July 2017, D.L. Liu & R.D. Liu XL2017267 (HMAS–L 140940); 39°28′22″ N 101°04′04″ E, 1563 m alt., on sand, 5 June 2018, D.L. Liu et al. ALS2018022 (HMAS–L 143912); 39°32′30″ N 101°06′34″ E, 1478 m alt., on sand, 5 June 2018, D.L. Liu et al. ALS2018040 (HMAS–L 153952).

**Notes:** This species is distinctive by having both uniseriate and (sub) biseriate ascospores arrangement, both laminal and marginal pycnidia, and a surrounding black area due to the aggregation of abundant pycnidia, which well-separate it from all the other *Placidium* species. *Placidium* is generally known as the only genus comprising species with laminal or marginal pycnidia [31]. Within this genus, very few species have both laminal and marginal pycnidia such as *Pl. velebiticum* (Zahlbr. ex Zschacke) Breuss [4,8]; however, *Pl. velebiticum* only has a uniseriate ascospores arrangement, which is different from *Pl. nigrum*.

**Figure 7:**
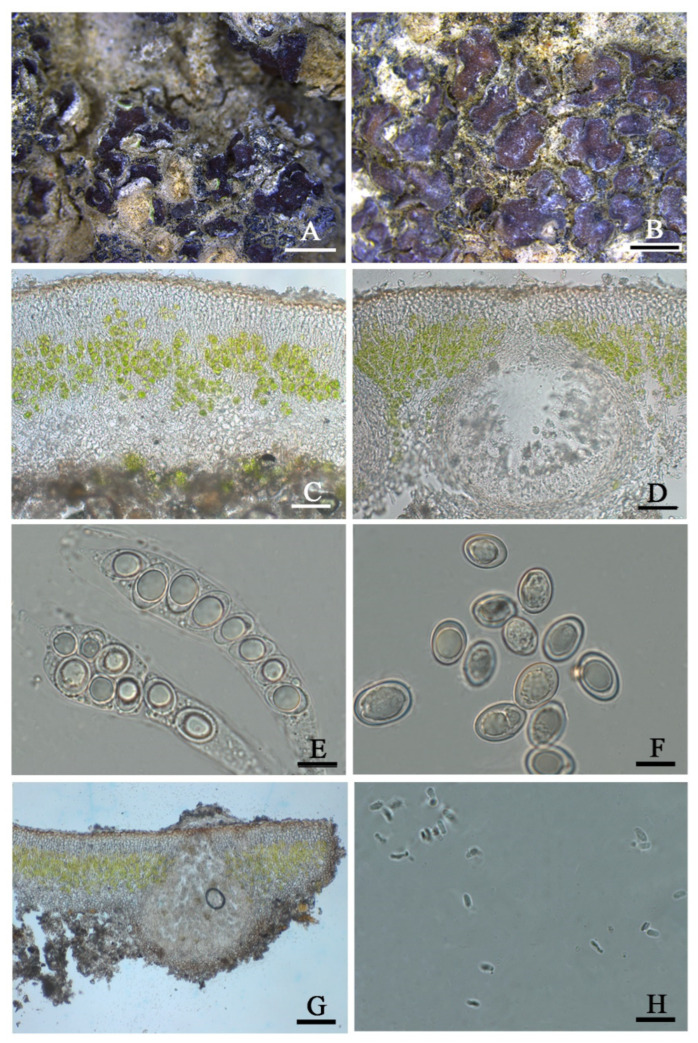
The thallus habit and the anatomic structure of *Placicium* *varium* sp. nov (holotype, HMAS–L 151970). (**A**). Squamulose thallus (HMAS–L 153948). (**B**). Squamulose thallus (HMAS–L 151967). (**C**). Thallus section. (**D**). Immersed perithecium. (**E**). Asci with biseriate and uniseriate ascospores. (**F**). Ascospores. (**G**). Pycnidium. (**H**). Conidia. Bars: (**A**,**B**) =2 mm, (**C**,**D**) =50 µm, (**E**,**F**) =10 µm, (**G**) =100 µm, and (**H**) =10 µm.

***Placidium* *varium*** T.T. Zhang & X.L. Wei, **sp. nov.** (Figure 7)

**Fungal Names No.:** FN571025

**Etymology:** The epithet ‘*varium*’ refers to the morphology and arrangement of the variable ascospores in this species.

**Typus:** CHINA. XINJIANG: Changji Hui Autonomous Prefecture, Qitai County. 44°13′32″ N 90°02′32″ E, 723 m alt., on the sand, 10 May 2021, X. Qian & T.T. Zhang 20210751 (holotype, HMAS–L 151970, nuLSU ON712841).

**Diagnosis:** This new species is characterized chiefly by both (sub)biseriate and uniseriate ascospores arrangement and having macro guttule in the center of each ascospore.

**Description:** Thallus squamulose, terricolous; lobes up to 4 mm wide, (183) 202 ± 10 (224) µm thick, deeply lobate, scattered or contiguous to densely aggregated, sometimes overlapped or imbricated, adpressed to the substrate or with raised margins free from the substrate, sometimes with a black margin; upper surface red-brown to dark brown, pruinose, dull; lower surface greyish brown; epinecral layer transparent, very thin, up to 20 µm thick; upper cortex (31) 46 ± 9 (65) µm thick, paraplectenchymatous cells (7.8) 8.2 ± 0.3 (8.6) µm diam, uppermost layer light brown, 10–13 µm thick; algal layer (73) 93 ± 14 (112) µm thick, algal cells globular, (7.2) 8.5 ± 0.9 (10) µm in diam., in clusters of 1–3 cells; medulla mixed type, (46) 54 ± 5(62) µm thick; lower cortex not delimited from the medulla, hyaline, (28) 33 ± 5.9 (43) µm thick, composed of spherical cells, (5.9) 7.7 ± 1.5 (10) µm in diam. Rhizohyphae (3.2) 4.4 ± 0.78 (5.7) µm thick, colorless. Pycnidia marginal, immersed, *Dermatocarpon*-type, subglobular to pyriform, up to 256–320 × 220–288 µm in size; conidia bacilliform to oblong-ellipsoid, (3.1) 3.5 ± 0.21 (3.9) × (1.0) 1.5 ± 0.2 (1.9) µm.

Perithecia immersed, subglobular to broad pyriform, up to 260 µm wide; perithecia wall bright, 19–25 µm thick; hymenium bright, 22–46 µm thick; involucrellum abscence; periphyses 18–26 µm long; asci cylindrical to clavate, 55–73 × 8–21 µm, 8 spored, uniseriate to (sub)biseriate, ascospores hyaline, ellipsoid to ovoid, (8.5) 11.8 ± 1.8 (15.3) × (7.6) 8.6 ± 1.0 (11.6) µm in size, central guttule (6.6) 8.0 ± 1.1 (10.8) × (4.9) 5.8 ± 0.68 (7.0) µm.

**Chemistry:** All the spot tests were negative, and no substances were detected by TLC.

**Ecology and distribution:** On the surface of sandy soil in the arid region of Xinjiang Autonomous, Northwest China. The specimens were collected at the bottom of a fixed sand area of cultivated shrubs. The habitat is a low-altitude open area with long daylight exposure, low precipitation and high evaporation. It has been known only in China up to now.

Additional specimens examined: CHINA. XINJIANG: Changji Hui Autonomous Prefecture, Fukang County. 44°22′59″ N 87°52′34″ E, 438 m alt., on the sand, 10 May 2021, X. Qian & T.T. Zhang 20210639 (HMAS–L 153949), 20210676 (HMAS–L 151953), 20210689 (HMAS–L 151955), 20210692 (HMAS–L 1519561), 20210698 (HMAS–L 151958), 20210696 (HMAS–L 151957); Wujiaqu City. 44°29′54″ N 87°28′21″ E, 408 m alt., on the sand, 9 May 2021, X. Qian & T.T. Zhang 20210576 (HMAS–L 153948), 20210599 (HMAS–L 151948), 20210608 (HMAS–L 151949), 20210611 (HMAS–L 151940), 20210623 (HMAS–L 151951); Qitai County. 44°13′32″ N 90°02′32″ E, 723 m alt., on the sand, 10 May 2021, X. Qian & T.T. Zhang 20210725 (HMAS–L 151969), 20210716 (HMAS–L 152826).

**Notes:** The ascospores arrangement is variable in this new species. Compared with other *Placidium* species with small ascospores, such as *Placidium arboretum* (Schwein. ex E. Michener) Lendemer (syn. *Placidium tuckermanii* (Rav. ex Mont.) Breuss), *Placidium andicola* (Breuss) Breuss, *Placidium chilense* (Breuss) Breuss, *Placidium corticola* (Räsänen) Breuss, and *Placidium ruiz-lealii* (Räsänen) Breuss, characterized by oval and uniseriate ascospores [30,32], the new species has more variable ascospores in shape and arrangement. Moreover, several species are not terricolous like this new species but corticolous in *Pl. corticola* and *Pl. arboretum* or saxicolous in *Pl. ruiz-lealii* [30]. Although *Pl. andicola* and *Pl. chilense* are terricolous, they are different from the new species in morphology besides the different shapes of ascospores; for example, *Pl. andicola* has brown but not red-brown thallus and only uniseriate ascospores in asci [30]. *Pl. chilense* has much more extensive (4–10 (−20) mm wide) and thicker (600 µm thick) lobes and much larger perithecia (up to 600 µm wide) than *Pl. varium* [30]. In the phylogenetic analyses, due to the absence of DNA sequences, among the species mentioned earlier related to *Pl. varium*, only *Pl. andicola* and *Pl. arboretum* can be further compared. It can be seen from the phylogenetic trees (Figure 2 and Figures S1–S3) *Pl. varium* clustered more closely to *Pl. andicola* than other species, although it is also pronounced there is a much distant relationship between these two species, and *Pl. varium* formed a well-supported separate clade, indicating it is a new species. As comparison, there is farther phylogenetic relationship between terricolous *Pl. varium* and corticolous *Pl. arboretum*, indicating the substrate type contributes to the species delimitation in *Placidium*.

## 4. Discussion

Nowadays, dry lands cover about 41% of Earth’s land surface and influence more than 38% of the global population [33]. Curbing the spread of land desertification in arid and semi-arid areas has become an urgent problem that needs to be focused on. Biological soil crusts (BSCs), as a ubiquitous phenomenon in these regions, play an essential role in soil nutrient cycling, sand stability, and hydrological processes [34]. When the BSCs developed to the lichen crust stage, they would contribute to the greater compressive strength and carbon and nitrogen fixation [35,36]. Lichens existing as the crust in the desert regions are very popular, such as *Endocarpon* and *catapyrenioid* lichens, which have started to be explored as a species diversity in China [37,38], but due to the small size of the lichen thallus and diverse, continuously changing, and transitional phenotypes, sometimes there will inevitably bring difficulties in correctly recognizing and choosing some related genera and species in the field survey, which then affects the sampling for the further study in the lab especially when we also should consider small sampling for not destroying too much the BSCs, all of which could lead to the missing findings for new taxa and the hidden species diversity in deserts.

Our phylogenetic analysis (Figure 2) well-supported *Clavascidium*, *Heteroplacidium*, and *Placidium* formed separate monophyletic clades, especially the genus *Heteroplacidium* situated in the outermost position, consistent with its only-exiting paraplectenchymatous medulla, while variable medulla types exited in *Clavascidium* and *Placidium*. *Clavascidium* and *Placidium* can be clearly distinguished by presence or absence of rhizines. Within the genus *Placidium*, it is generally known only uniseriate ascospores arrangement existed [8,9], but sometimes, both uniseriate and biseriate ascospores arrangements co-existed in some species such as *Placidium acarosporoides* (Zahlbr.) Breuss [30] and two new species *Pl. nigrum* and *Pl. varium*, indicating uniseriate and biseriate ascospores arrangements are continuously changing character between *Clavascidium* and *Placidium*, and within each of these two genera; nevertheless, a single biseriate ascospores arrangement was not seen in *Placidium.* As comparison, within the genus *Clavascidium*, it is generally known only biseriate ascospores arrangement existed [6,8,9], but sometimes, both uniseriate and biseriate ascospores arrangements also coexisted in some species such as *Cl. pseudorufescens* and *Cl. semaforonense* [30] and *Cl. lacinulatum*; however, single a uniseriate ascospores arrangement was not seen in *Clavascidium*. Laminal pycnidia are almost an exclusive character in most genera of catapyrenioid lichens, except *Placidium* with laminal or marginal pycnidia; however, marginal pycnidia were also found in *Cl. semaforonense* [30] and new species *Cl. sinensel*; further supporting pycnidia position is also a continuously changing character between *Clavascidium* and *Placidium*. The only difference in the pycnidia position between *Clavascidium* and *Placidium* is both laminal and marginally coexisted in some species of *Placidium* but not in *Clavascidium*. Besides, some new characters have been put forward to define species in *Placidium*, well-supported by the phylogenetic analysis, such as aggregated pycnidia and a glossy upper surface.

This study found that *Clavascidium* and *Placidium* are distributed in Northwest China, where are harsh environments such as drought and oligotrophic. The previous studies showed that ecological environments could influence the lichen distribution, similar to *Placidium* [8,39]; therefore, the relationship between the adaptative characters and distribution should be paid more attention. The finding of the new species *Cl. sinense* provides a further clue to reconsider the generic relationship between *Clavascidium* and *Placidium*. Moreover, the coexistence of uniseriate and biseriate ascospores arrangement may promote the chance of ascospores discharge for better reproduction. *Pl. nitidulum* only grows at high altitudes above 3000 m alt., and the adaptive phenotype may have produced, for example, tiny lobes and thick algal layer may increase photosynthetic efficiency, and the developed paraphyses in the perithecia would be helpful in squeezing the asci for a better discharge of ascospores through swelling caused by absorbing water. *Pl.* *nigrum* has a relatively wide ecological niche, ranging from low altitude to high altitude (400–3500 m alt.), with the climate type from arid to wet (90–523 mm annual precipitation) [40]; we hypothesize it is so abundant, pycnidia could help itself to propagate in the variable environments through its asexual reproduction. *Pl. varium* grows in the Xinjiang Autonomous Region dry area with annual precipitation of 180–270 mm; small size and macro guttule of the ascospores may decrease their weight and correspondingly increase their propagative velocity for colonizing a farther and broader living space. However, whether these phenotypic characters are significantly related to the adaptive mechanism need to be further studied.

Therefore, to better recognize the species diversity, understand the adaptive phenotype and other mechanism, and explore more potential species resources that could be applied to restrain sustainable desertification; more taxonomic studies should be continuously carried out on the BSCs-related taxa, such as catapyrenioid lichens, and genomic-scale adaptive evolution studies also need to be explored in the near future.

## Figures and Tables

**Table 1:** Key to the species of *Clavascidium* and *Placidium* in China.

1. Presence of rhizines	2
1′. Absence of rhizines	3
2. Upper cortex thinner 50–70 µm; medulla paraplectenchymatous	*Cl. lacinulatum*
2′. Upper cortex thicker 60–100 µm; medulla mixed type	*Cl. sinense*
3. Pycnidia both marginal and laminal	*Pl.* *nigrum*
3′. Pycnidia either marginal or laminal	4
4. Pycnidia laminal	5
4′. Pycnidia marginal	6
5. Squamules tiny 0.7–2 mm; asci slender 45–65 × 5–8 µm; medulla paraplectenchymatous	*Pl. nitidulum*
5′. Squamules larger 2–7 mm; asci 70–90 × 10–15 µm; medulla mixed type	*Pl. squamulosum*
6. Ascospores more elliptical (12–17 × 5.5–7 μm); squamules margin hairy	*Pl. pilosellum*
6′. Acospores are more rounded (10–13.5 × 7.5–9.5 μm) with central guttule; squamules margin smooth	*Pl. varium*

## Data Availability

Publicly available datasets were analyzed in this study. All newly generated sequences were deposited in GenBank (https://www.ncbi.nlm.nih.gov/genbank/; Table S1, accessed on 4 June 2022). All new taxa were deposited in Fungal names (https://www.fungalinfo.im.ac.cn, accessed on 8 June 2022).

## Supplementary Materials

The following supporting information can be downloaded at https://www.mdpi.com/article/10.3390/jof8070729/s1: Table S1: Details of specimens (species name, voucher information, and GenBank numbers) used in this study. Figure S1: The Bayesian tree based on the concatenated ITS + nuLSU (two genes) data set. Figure S2: The maximum-likelihood tree based on the ITS data set. Figure S3: The maximum-likelihood tree based on the nuLSU data set.
