# Supplementary material for: Hidden Species Diversity was Explored in Two Genera of Catapyrenioid Lichens (Verrucariaceae, Ascomycota) from the Deserts of China
Source: J Fungi (Basel). 2022 Jul 13;8(7):729. doi: 10.3390/jof8070729 (PMC9319096; doi:10.3390/jof8070729)
Supplement: Supplementary file 1 [file jof-08-00729-s001.zip › jof-1794430-supplementary.pdf]

Table S1. Specimens used for DNA extraction and GenBank accession number of all samples used in this study

Species	Voucher information	GenBank Accession numbers	
		ITS	nu LSU
C. lacinulatum 1	—	—	EF643762
C. lacinulatum 2	—	EF469155	EF469158
C. lacinulatum 3	M. Prieto 552	GU228960	GU228913
C. lacinulatum	**20210302 (HMAS-L 153959)**	**ON685892**	**ON678147**
C. lacinulatum	**20210307 (HMAS-L 153961)**	**ON685893**	**ON678148**
C. lacinulatum	**20210274 (HMAS-L 153956)**	**ON685894**	**ON678146**
C. lacinulatum	**20210499 (HMAS-L 153954)**	**ON685895**	**ON678150**
C. lacinulatum	**20210436 (HMAS-L 151942)**	**ON685896**	**ON678149**
C. lacinulatum	**20201529 (HMAS-L 153781)**	**ON685897**	—
C. lacinulatum	**20210273 (HMAS-L 153955)**	**ON685898**	—
C. lacinulatum	**20210283 (HMAS-L 153957)**	**ON685899**	—
C. lacinulatum	**20210299 (HMAS-L 153962)**	**ON685900**	—
C. lacinulatum	**20210300 (HMAS-L 153958)**	**ON685901**	—
C. lacinulatum	**20210306 (HMAS-L 153960)**	**ON685902**	—
C. lacinulatum	**QX20200026 (HMAS-L 153953)**	**ON685903**	—
C. lacinulatum var. *atrans* 1	M. Prieto 1703	GU228957	GU228910
C. lacinulatum var. *atrans* 2	M. Prieto 1702	GU228958	GU228912
C. lacinulatum var. *erythrostratum*	ARIZ 551162	GU228965	—
C. aff. *lacinulatum* var. *latisporum*	M. Prieto 1704	GU228959	GU228911
C. lacinulatum var. *latisporum* 1	ARIZ 551304	—	GU228933
C. lacinulatum var. *latisporum* 2	ARIZ 551314	—	GU228934
C. pseudorufescens 1	LI 127885	GU228963	GU228945
C. pseudorufescens 2	LI 566404	GU228966	GQ344563
C. semaforonense	M. Prieto 63	GU228961	GU228930
***C. sinense* sp. nov.**	**20210245 (HMAS-L 153946)**	**ON712842**	**ON712829**
***C. sinense* sp. nov.**	**20210246 (HMAS-L 153947)**	**ON712843**	**ON712830**
Placidium acrosporoides	—	—	EF643760
P. adami-borosi 1	Aptroot S/N	GU228985	GU228936
P. adami-borosi 2	LI 285591	GU228986	GU228942
P. aff. *andicola*	LI 512226	GU228983	GU228941
P. arboreum 1	COLO 479327	GU228995	—
P. arboreum 2	AFTOL-ID 2285	—	EF643765
P. arboreum 3	CG 579	KY769559	—
P. deosaiense 1	LAH36819	MW653287	MW653270
P. deosaiense 2	LAH36822	MW653290	MW653273
P. fingens	LI 271006	GU228989	GU228935
P. imbecillum 1	LI 364261	GU228979	GU228940
P. imbecillum 2	M. Prieto 664	GU228980	—
P. aff. *lachneoides*	M. Prieto 564	GU228972	GU228906
P. lachneum 1	Leavitt 18-619	MZ244187	—
P. lachneum 2	Leavitt 18-577	MZ244185	—
P. lachneum 3	M. Prieto 322	—	GU228926

P. lachneum 4	AFTOL-ID 2286	—	EF643761
P. lachneum var. *oleosum*	M. Prieto 105	GU228981	GU228929
***P. nitidulum* sp. nov.**	**20210560 (HMAS-L 151947)**	**ON712844**	**ON712835**
***P. nitidulum* sp. nov.**	**20210552 (HMAS-L 151946)**	**ON712845**	**ON712833**
***P. nitidulum* sp. nov.**	**20210421 (HMAS-L 151962)**	**—**	**ON712834**
P. norvegicum	M. Prieto 330	GU228973	GU228904
P. pilosellum 1	M. Prieto 439	GU228968	GU228907
P. pilosellum 2	M. Prieto 3	GU228993	GU228925
P. pilosellum 3	M. Prieto 128	GU228967	—
P. podolepis	LI 297365	GU228956	GU228917
P. rufescens 1	M. Prieto 10	GU228970	GU228931
P. rufescens 2	M. Prieto 164	GU228971	GU228914
P. squamulosum 1	M. Prieto 339	—	GU228928
P. squamulosum 2	M. Prieto 336	GU228994	—
P. squamulosum var. *argentinum*	M. Prieto 1705	GU228991	GU228922
P. subrufescens	Aragón 33/04	GU228974	GU228903
P. tenellum	MA 16300	—	GQ344562
***P. tumidulum* sp. nov.**	**20210535 (HMAS-L 153951)**	**ON712848**	**ON712831**
***P. tumidulum* sp. nov.**	**20210524 (HMAS-L 151966)**	**ON712849**	**ON712832**
***P. tumidulum* sp. nov.**	**20210606 (HMAS-L 151967)**	**ON712858**	**ON712836**
***P. tumidulum* sp. nov.**	**20210523 (HMAS-L 151965)**	**ON712852**	**—**
***P. tumidulum* sp. nov.**	**20201384 (HMAS-L 153732)**	**ON712846**	**—**
***P. tumidulum* sp. nov.**	**20201486 (HMAS-L 153786)**	**ON712847**	**—**
***P. tumidulum* sp. nov.**	**20210666 (HMAS-L 151952)**	**ON712850**	**—**
***P. tumidulum* sp. nov.**	**20210516 (HMAS-L 151944)**	**ON712851**	**—**
***P. tumidulum* sp. nov.**	**20210522 (HMAS-L 151945)**	**ON712857**	**—**
***P. tumidulum* sp. nov.**	**20210534 (HMAS-L 153950)**	**ON712853**	**—**
***P. tumidulum* sp. nov.**	**20210461 (HMAS-L 151963)**	**ON712860**	**—**
***P. tumidulum* sp. nov.**	**20210746 (HMAS-L 151941)**	**ON712859**	**—**
***P. tumidulum* sp. nov.**	**XL2017267 (HMAS-L 140940)**	**ON712856**	**—**
***P. tumidulum* sp. nov.**	**ALS2018040 (HMAS-L 153952)**	**ON712855**	**—**
***P. tumidulum* sp. nov.**	**ALS2018022 (HMAS-L 143913)**	**ON712854**	**—**
P. umbrinum 1	LI 350628	GU228962	GU228924
P. umbrinum 2	AFTOL-ID 2274	—	EF643749
***P. varium* sp. nov.**	**20210692 (HMAS-L 151956)**	**ON712861**	**ON712838**
***P. varium* sp. nov.**	**20210698 (HMAS-L 151958)**	**ON712863**	**ON712839**
***P. varium* sp. nov.**	**20210725 (HMAS-L 151969)**	**ON712864**	**ON712840**
***P. varium* sp. nov.**	**20210696 (HMAS-L 151957)**	**ON712862**	**—**
***P. varium* sp. nov.**	**20210576 (HMAS-L 153948)**	**ON712865**	**—**
***P. varium* sp. nov.**	**20210689 (HMAS-L 151955)**	**ON712866**	**—**
***P. varium* sp. nov.**	**20210676 (HMAS-L 151953)**	**ON712867**	**—**
***P. varium* sp. nov.**	**20210639 (HMAS-L 153949)**	**ON712868**	**—**
***P. varium* sp. nov.**	**20210599 (HMAS-L 151948)**	**ON712869**	**—**
***P. varium* sp. nov.**	**20210608 (HMAS-L 151949)**	**ON712870**	**—**
***P. varium* sp. nov.**	**20210623 (HMAS-L 151951)**	**ON712871**	**—**

***P. varium* sp. nov.**	**20210716 (HMAS-L 152816)**	**ON712872**	—
***P. varium* sp. nov.**	**20210611 (HMAS-L 151940)**	**ON712873**	—
***P. varium* sp. nov.**	**20210751 (HMAS-L 151970)**	—	**ON712841**
P. velebiticum 1	LI 297363	GU228975	GU228921
P. velebiticum 2	LI 285722	GU228977	GU228938
P. yoshimurae	LI 316277	GU228984	GU228905
Heteroplacidium acervatum 1	LI 271015	GU228954	GQ344564
H. acervatum 2	M. Prieto 399	GU228955	GU228932
H. compactum 1	M. Prieto 1607	GU228949	GU228916
H. compactum 2	M. Prieto 1701	GU228952	GU228918
H. congeatuum 1	LI 552268	GU228950	GU228920
H. congeatuum 2	LI 297536	GU228951	GU228919
H. contumescens	—	—	EF643755
H. divisum	LI 218468	GU228953	GU228915
H. fuscula	AFTOL-ID 2255	—	EF643793
H. imbricatum	—	—	EF643756
Placopyrenium bucekii 1 (outgroup)	—	EU010245	EF643767
Pl. bucekii 2 (outgroup)	—	EU010246	EF643768
Pl. canellum 1 (outgroup)	—	—	EF643784

Notes: Newly generated sequences are in bold font. '—' indicates that the corresponding information or sequence is absent.

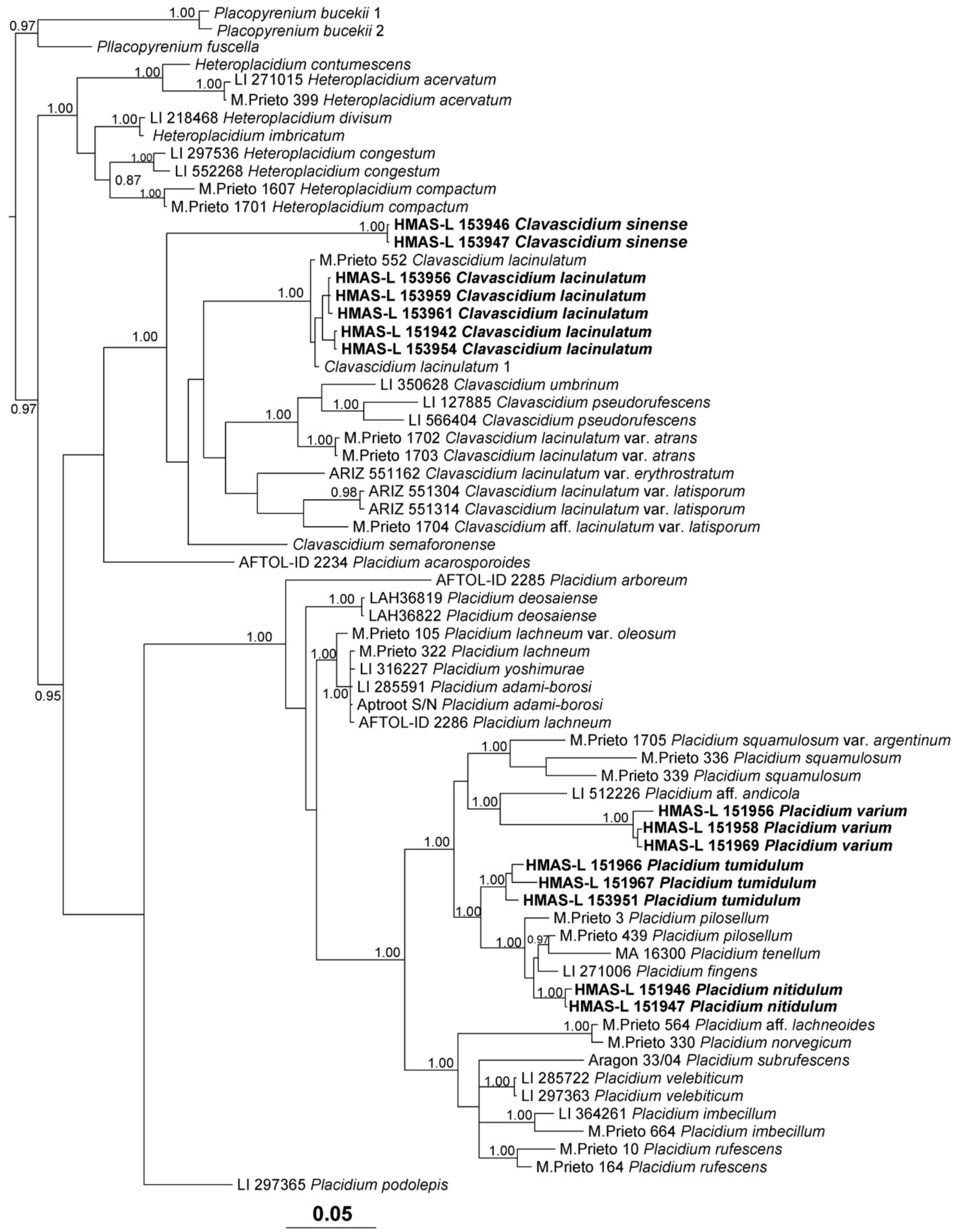

Figure S1. The Bayesian tree based on the concatenated ITS + nuLSU data sets. The numbers in each node represent posterior probability (PP) values. PP values ≥ 0.95 were plotted on the branches of the tree. The samples in bold indicate that these sequences were newly generated for this study. Scale in 0.05 substitution per site.

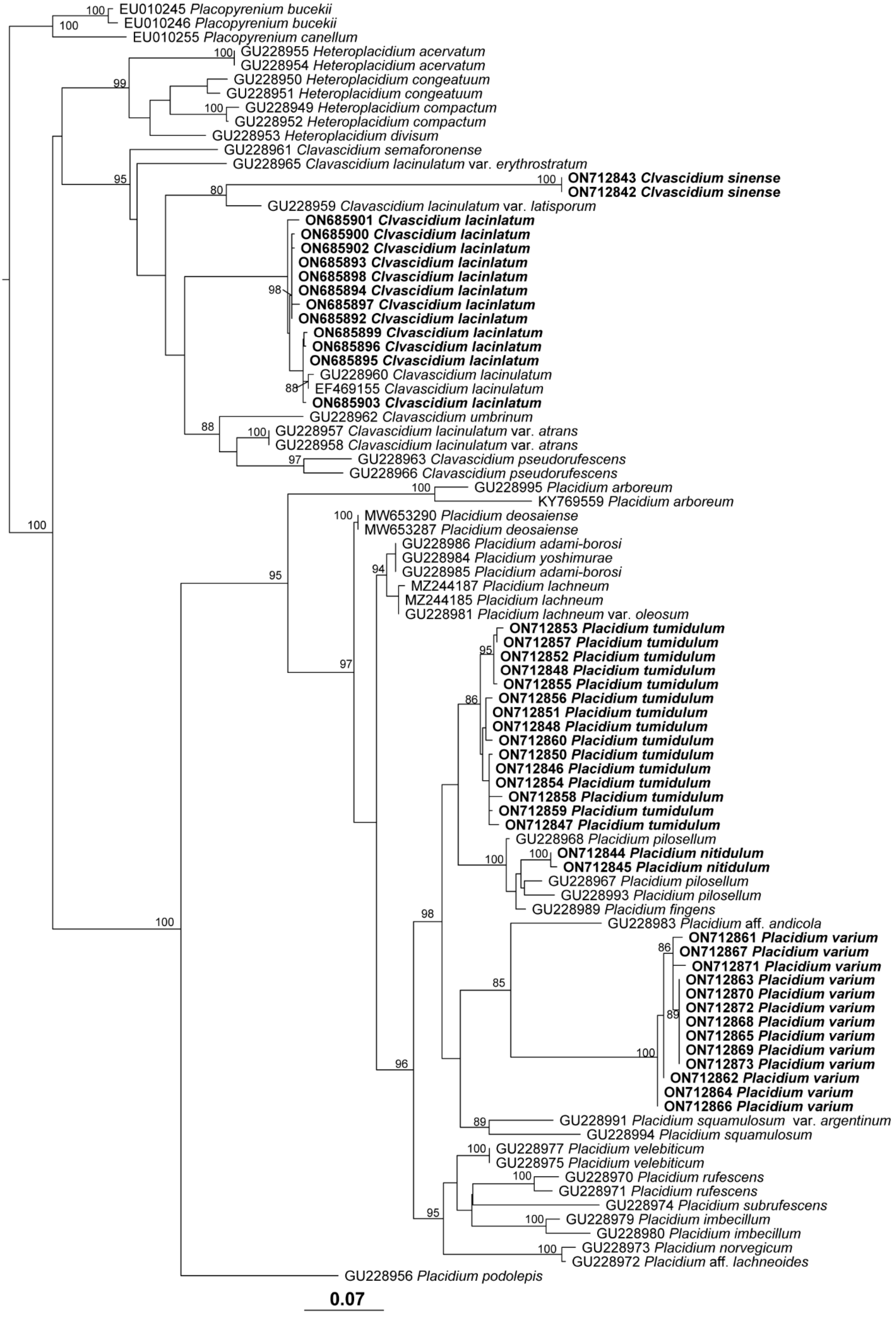

Figure S2. The maximum likelihood tree based on the ITS data set. The numbers in each node represent bootstrap support (BS)

values. BS values ⩾ 75% were plotted on the branches of the tree. The samples in bold indicate that these sequences were newly generated for this study. Scale in 0.07 substitution per site.

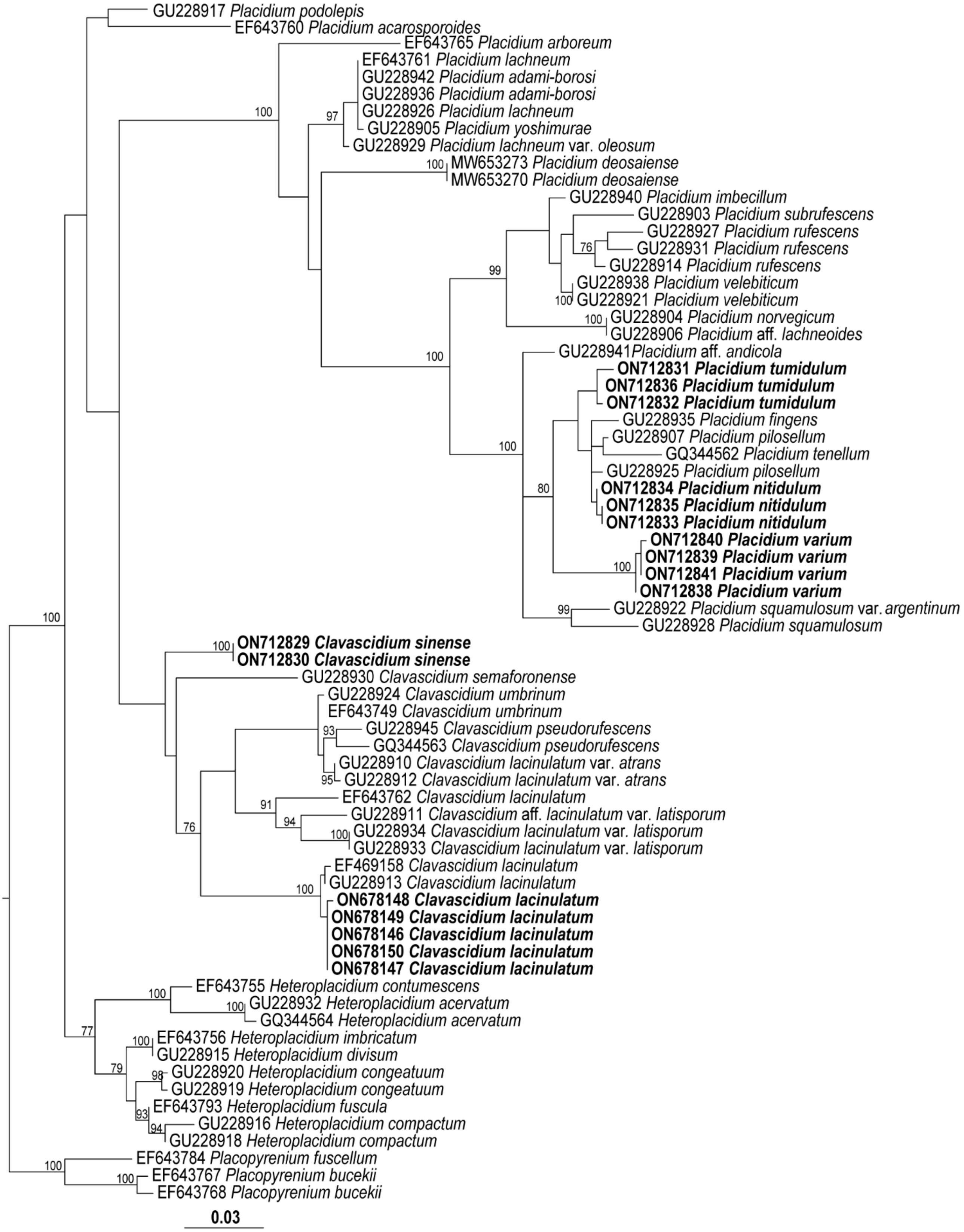

Figure S3. The maximum likelihood tree based on the LSU data set. The numbers in each node represent bootstrap support (BS) values. BS values ⩾ 75% were plotted on the branches of the tree. The samples in bold indicate that these sequences were newly generated for this study. Scale in 0.03 substitution per site.
